# Association of glomerular DNA damage and DNA methylation with one-year eGFR decline in IgA nephropathy

**DOI:** 10.1038/s41598-019-57140-0

**Published:** 2020-01-14

**Authors:** Kaori Hayashi, Akihito Hishikawa, Akinori Hashiguchi, Tatsuhiko Azegami, Norifumi Yoshimoto, Ran Nakamichi, Hirobumi Tokuyama, Hiroshi Itoh

**Affiliations:** 1 Department of Internal Medicine, School of Medicine, Keio University, Tokyo, Japan; 2 Department of Pathology, School of Medicine, Keio University, Tokyo, Japan

**Keywords:** Diseases, Medical research, Nephrology

## Abstract

Accumulation of DNA double-strand breaks (DSBs) is linked to aging and age-related diseases. We recently reported the possible association of DNA DSBs with altered DNA methylation in murine models of kidney disease. However, DSBs and DNA methylation in human kidneys was not adequately investigated. This study was a cross-sectional observational study to evaluate the glomerular DNA DSB marker γH2AX and phosphorylated Ataxia Telangiectasia Mutated (pATM), and the DNA methylation marker 5-methyl cytosine (5mC) by immunostaining, and investigated the association with pathological features and clinical parameters in 29 patients with IgA nephropathy. To evaluate podocyte DSBs, quantitative long-distance PCR of the nephrin gene using laser-microdissected glomerular samples and immunofluorescent double-staining with WT1 and γH2AX were performed. Glomerular γH2AX level was associated with glomerular DNA methylation level in IgA nephropathy. Podocytopathic features were associated with increased number of WT1(+)γH2AX(+) cells and reduced amount of PCR product of the nephrin gene, which indicate podocyte DNA DSBs. Glomerular γH2AX and 5mC levels were significantly associated with the slope of eGFR decline over one year in IgA nephropathy patients using multiple regression analysis adjusted for age, baseline eGFR, amount of proteinuria at biopsy and immunosuppressive therapy after biopsy. Glomerular γH2AX level was associated with DNA methylation level, both of which may be a good predictor of renal outcome in IgA nephropathy.

## Introduction

Various stresses, including UV radiation, chemicals, reactive oxygen species (ROS), DNA replication errors and mechanical stress, cause DNA damage^1^. Although various types of DNA damage have been reported, DNouble-strand breaks (DSBs) are biologically important because of the repair difficulty^2^. Accumulation of DNA damage is linked to aging and age-related diseases, such as cancer. Recent studies reported that increased DNA damage and reduced DNA repair capacity also play roles in the pathogenesis of cardiovascular and metabolic diseases^3^. Angiotensin II and aldosterone are key factors in kidney disease that cause DNA damage, such as DSBs, and DNA base modification, such as 8-OHdG^4,5^. However, the association of DNA DSBs in human kidneys with clinical parameters and pathological findings has not been adequately elucidated.

Previous *in vitro* studies suggested that DNA DSBs and their repair processes induced altered DNA methylation^6–8^. We recently investigated the association of DNA DSBs with altered DNA methylation status in glomerular podocytes using murine models of diabetic nephropathy^9^. Increased DNA DSBs in podocytes due to both decreased levels of the DNA repair factor KAT5 and increased DNA damage induced by high-glucose conditions increased DNA methylation in the nephrin promoter region. However, the association of DNA DSBs with DNA methylation status in human kidneys was not investigated.

IgA nephropathy is the most common glomerulonephritis in many countries, especially countries in Asia. The prognosis of IgA nephropathy is not optimistic because 20–40% of patients progress to end-stage renal disease (ESRD) within 20 years of diagnosis^10,11^. The Oxford classification of IgA nephropathy is widely used in clinical practice to evaluate renal damage associated with renal outcome^12,13^. Recent studies have indicated that podocyte damage plays a major role in the progression of IgA nephropathy^14^, and podocytopathic features, which are indicated as podocyte hypertrophy or sclerosis at the tubular pole, must be described in scoring using the revised Oxford Classification from 2016^13^. Therefore, evaluations of podocyte DSBs are important to determine whether they are involved in disease progression partially via epigenetic changes. The association of glomerular DNA DSBs and DNA methylation with disease progression has not been shown in IgA nephropathy, although a previous study reported an association between 8-OHdG, an indicator of oxidative DNA damage, and interstitial fibrosis in IgA nephropathy^15^.

This study investigated the association between glomerular γH2AX immunostaining, which are markers of DNA DSBs, and glomerular 5-methyl cytosine (5mC) immunostaining, a marker of DNA methylation. γH2AX, the phosphorylated histone variant of H2AX, plays an important role in the recruitment of DNA repair factors to damaged sites during the initial phase of repair, and it is frequently used as a marker of DNA DSBs^16^. We confirmed glomerular DNA DSBs using immunostaining with phosphorylated ataxia telangiectasia mutated (pATM), activated ATM that phosphorylates H2AX in the response to DNA DSBs. To evaluate podocyte DNA DSBs, the amount of DNA DSBs in the nephrin gene, including the promoter region, was examined by quantitative PCR, as previously described in mice^9,17^, using laser-microdissected glomerular samples as well as double-immunostaining with podocyte marker WT1 and γH2AX. The PCR product levels reflect DNA DSBs primarily in podocytes because opened chromatin is vulnerable to injury^18–20^, and chromatin condensation of the nephrin promoter region is opened in podocytes^9^. The present study revealed an association of glomerular DNA DSBs with DNA methylation and eGFR decline over one year in IgA nephropathy patients.

## Results

### Participant characteristics

A total of 29 individuals (17 males and 12 females) aged 45.7 ± 3.0 years were eligible for inclusion in the present study. Table 1 shows the general characteristics of the subjects. The following average clinical values were recorded: eGFR at biopsy was 57.0 ± 4.4 ml/min/1.73 m^2^; eGFR 1 year after biopsy was 55.7 ± 4.9 ml/min/1.73 m^2^; the amount of proteinuria at biopsy was 1.76 ± 0.30 g/day; and the amount of proteinuria 1 year after biopsy was 0.55 ± 0.13 g/gCr. Four control samples with normal glomerular findings from the patients with asymptomatic microscopic hematuria or with normal urinalysis were available in this study to compare the levels of DNA DSBs and DNA methylation with IgA nephropathy kidneys. As shown in Table 1, eGFR at baseline and one year after biopsy were significantly reduced in IgA nephropathy patients compared with the controls. In IgA nephropathy patients, 2, 7, 23, and 11 patients showed mesangial hypercellularity, endocapillary hypercellularity, segmental sclerosis and crescents, respectively, according to the Oxford classification. Considering tubular atrophy and interstitial fibrosis, 6 and 3 patients were classified into T1 and T2, respectively. Podocytopathy was observed in 9 patients. Fifteen patients received immunosuppressive therapy. Seventeen patients exhibited hypertension at biopsy using the criteria described in the Methods section, and 15 patients were treated with angiotensin receptor blocker (ARB).

**Table 1:** Baseline characteristics of all participants.

Clinical parameters	IgAN patients	Controls	p value
Age (years)	45.7 ± 3.0	32.8 ± 8.0	p = 0.12
Sex (male/female)	17/12	1/3	p = 0.20
Systolic blood pressure (mmHg)	127 ± 18	116 ± 11	p = 0.25
Diastolic blood pressure (mmHg)	77 ± 16	72 ± 16	p = 0.60
eGFR at biopsy^A^ (ml/min/1.73 m^2^)	57.0 ± 4.4	87.8 ± 11.6	p = 0.03
eGFR 1 year after biopsy^B^ (ml/min/1.73 m^2^)	55.7 ± 4.9	90.5 ± 12.5	p = 0.03
Proteinuria at biopsy (g/day)	1.76 ± 0.30	—	
Proteinuria 1 year after biopsy (g/gCr)	0.55 ± 0.13	—	
Diabetes at biopsy (%)	1 (3)	0 (0)	
Hypertension at biopsy (%)	17 (59)	0 (0)	
Immunosuppressive therapy after biopsy (%)	15 (52)	—	
Use of Angiotensin Receptor Blocker at biopsy (%)	15 (52)	—	
**Pathological features in IgA nephropathy patients**			
The number of global sclerosis/the number of total glomeruli	0.24 ± 0.04		
Oxford Classification			
Mesangial hypercellularity (M0/M1)	27/2		
Endocapillary hypercellularity (E0/E1)	22/7		
Segmental glomerulosclerosis (S0/S1)	6/23		
Tubular atrophy/interstitial fibrosis (T0/T1/T2)	20/6/3		
Crescent (C0/C1)	18/11		
Podocytopathic features (%)	9 (32)		

IgAN, IgA nephropathy; eGFR, estimated glomerular filtration rate.

### Association of DNA double-strand breaks with glomerular DNA methylation in patients with IgA nephropathy

**Figure 1:**
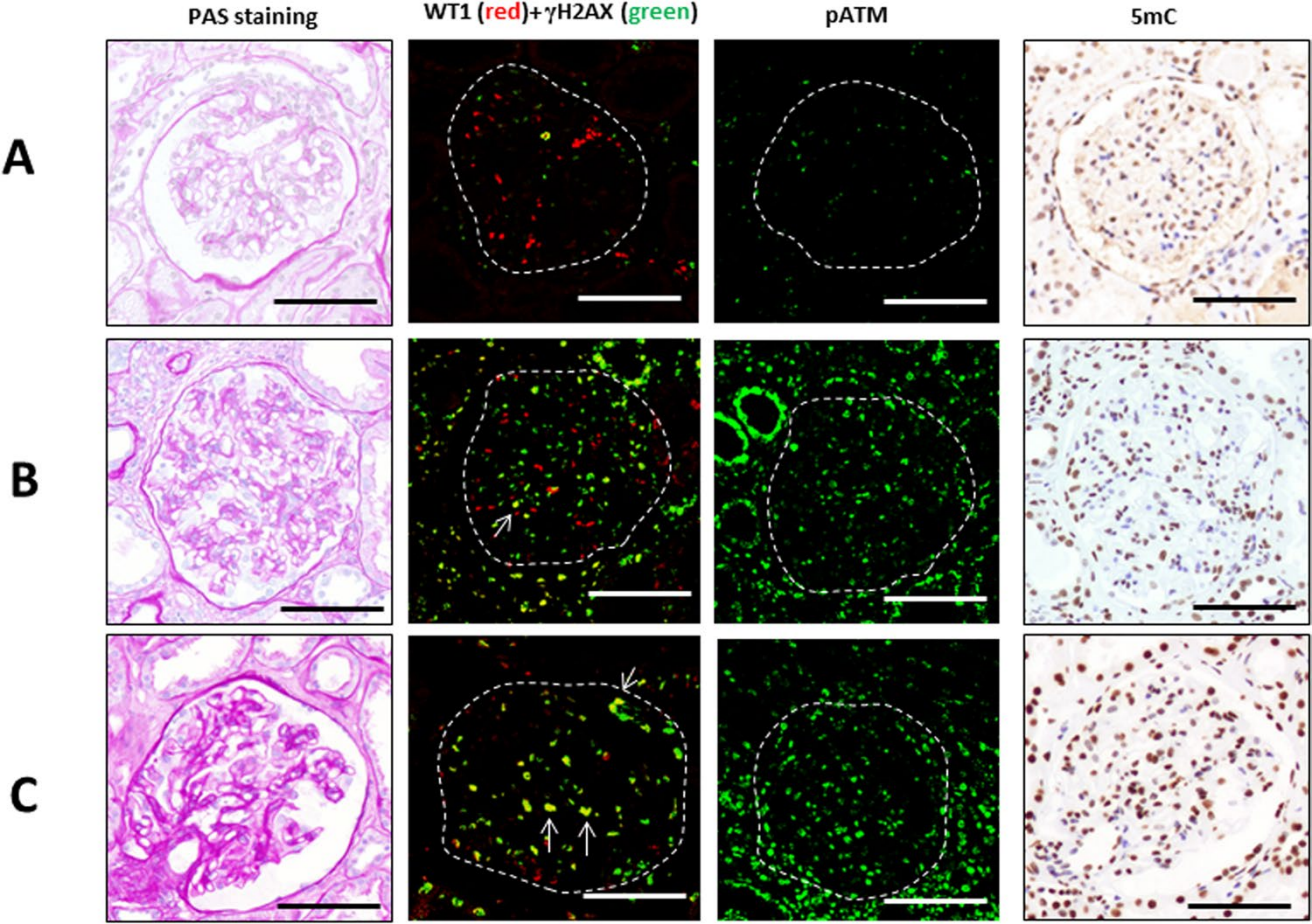
Immunostaining of γH2AX, WT1 and 5mC in patients with IgA nephropathy and controls. Examples of PAS staining and immunostaining with γH2AX (green) and WT1 (red), pATM and 5mC in glomeruli of IgA nephropathy and controls. (**A**) A control kidney sample of 44-year-old female, (**B**) 65-year-old male of IgA nephropathy without podocytopathic features and (**C**) 55-year-old male of IgA nephropathy with podocytopathic features. Arrows indicate γH2AX and WT1 double-positive cells. Scale bars: 50 μm.

First, DNA DSB and DNA methylation levels were examined in glomeruli of IgA nephropathy patients and controls. Immunostainings of γH2AX and pATM, DNA DSB markers, and 5-methyl cytosine (5mC), a DNA methylation marker, were observed in the nuclei of glomerular and tubular cells as shown in Fig. 1. The number of mean γH2AX-positive cells and pATM-positive cells per glomerulus, which indicate DNA DSBs, were significantly increased in patients with IgA nephropathy compared to controls (p = 0.0015 and p = 0.0060, respectively).

To evaluate podocyte DNA DSBs in IgA nephropathy, immunofluorescent double staining with γH2AX and podocyte marker WT1, and a long-distance PCR analysis with samples obtained from serial biopsy slides using laser microdissection were performed. The number of glomerular WT1(+)γH2AX(+)cells, which indicate podocytes with DNA DSBs, was increased in IgA nephropathy compared to controls (p = 0.0024). The numbers of glomerular γH2AX-positive cells was correlated with the number of WT1(+)γH2AX(+) cells (r = 0.3465, p = 0.0482), suggesting that podocyte DNA DSBs were associated with increased glomerular DSBs at least in part. Then, we prepared primer sets in the nephrin genes to quantify DNA DSBs in the region. The amount of PCR products of the nephrin gene adjusted by the total glomerular area was correlated with the mean number of WT1(+)γH2AX(+) cells per glomerulus (r = −0.4244, p = 0.0274).

**Figure 2:**
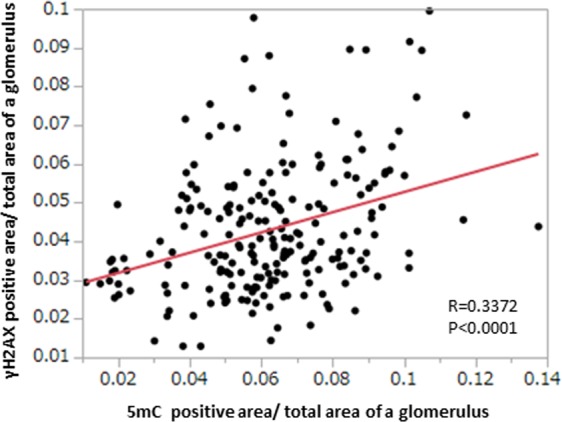
Association of DNA double-strand breaks with glomerular DNA methylation. The immunostaining level of γH2AX was positively correlated with that of 5mC.

Next, we investigated the association between DNA DSBs and DNA methylation in 243 glomeruli of IgA nephropathy using γH2AX and 5mC immunostaining by Pearson’s correlation coefficient and a univariate regression model. The glomerular γH2AX level was significantly correlated with the 5mC level (r = 0.3372, p < 0.0001) (Fig. 2). The mean number of γH2AX-positive cells per glomerulus was significantly correlated with the γH2AX level (r = 0.4983, p = 0.0059), and also associated with the 5mC level (r = 0.3738, p = 0.0458).

### Association of DNA double-strand breaks and DNA methylation with the pathological features of IgA nephropathy

**Figure 3:**
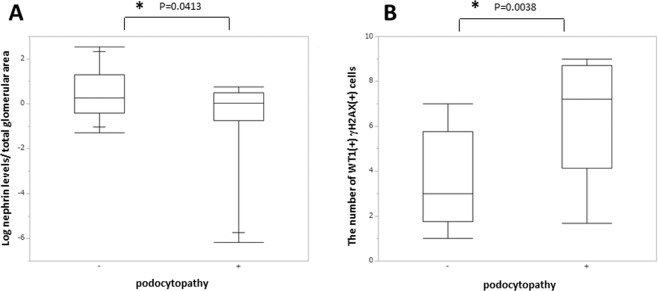
Podocyte DNA DSBs were associated with podocytopathic features. (**A**) The log nephrin/total glomerular area and (**B**) the number of WT1(+)γH2AX (+) cells were associated with podocytopathic features in patients with IgA nephropathy.

We examined the association of glomerular γH2AX and 5mC levels and long-distance PCR products of the nephrin gene with the pathological features of IgA nephropathy. The glomerular γH2AX level was marginally associated with segmental sclerosis in the analysis of each glomerulus (p = 0.07). No significant association was observed with glomerular γH2AX staining of a specific glomerular area, such as vascular or tubular poles. Presence of podocytopathy was significantly associated with the long-PCR product of the nephrin gene (p = 0.0413) as well as the mean number of WT1(+)γH2AX(+) cells per glomerulus (p = 0.0038) (Fig. 3A,B).

### Association of DNA double-strand breaks and DNA methylation with the eGFR decline in patients with IgA nephropathy

The association between eGFR decline over one year after biopsy and the mean glomerular γH2AX and 5mC levels were investigated using a multiple linear regression model adjusted for age, eGFR and urinary protein level at biopsy (Table 2). Glomerular γH2AX and 5mC levels were significantly associated with increased slope of eGFR decline over one year independently of age, eGFR and amount of proteinuria at biopsy (r^2^ = 0.41, p = 0.0068 and r^2^ = 0.52, p = 0.0005, respectively). In addition, the glomerular γH2AX and 5mC levels were also significantly associated with the slope of eGFR decline in multiple regression analysis adjusted for age, eGFR amount of proteinuria at biopsy and presence of immunosuppressive therapy or ARB treatment (γH2AX: r^2^ = 0.43, p = 0.0060 and r^2^ = 0.41, p = 0.0091, 5mC: r^2^ = 0.60, p = 0.0001 and r^2^ = 0.55, p = 0.0005, respectively). We confirmed that the numbers of γH2AX-positive cells or pATM-positive cells were correlated with the slope of eGFR decline over one year (r = −0.7116, p < 0.0001; r = −0.4243, p = 0.0307). Multivariate regression analysis showed the significant association of the number of γH2AX-positive cells with the slope of eGFR decline after adjustment by age, baseline eGFR and proteinuria (r^2^ = 0.58, p = 0.00014). It was also significant after adjustment by age, sex, baseline eGFR, proteinuria and presence of hypertension (r^2^ = 0.62, p = 0.00009).

**Table 2:** Multiple regression analysis of the slope of eGFR decline over one year.

	Glomerular γH2AX		Glomerular 5mC	
	Coefficient (95% CI) (per 10% of total glomerulus)	p value	Coefficient (95% CI) (per 10% of total glomerulus)	p value
Model A	−29.35 (−49.68 to −9.02)	p = 0.0068	−38.73 (−58.59 to −18.86)	P = 0.0005
Model B	−30.78 (−51.72 to −9.84)	P = 0.0060	−45.41 (−65.58 to −25.24)	P = 0.0001
Model C	−30.33 (−52.28 to −8.37)	P = 0.0091	−39.48 (−59.41 to −19.55)	P = 0.0005

CI: confidence interval. Glomerular γH2AX or 5mC were calculated by total γH2AX or 5mC-positive area/total glomerular area in each patient. Model A: multiple linear regression model for eGFR decline over one year (eGFR 1 year after biopsy- eGFR at biopsy, ml/min/1.73 m^2^) adjusted by the factors, including age, baseline eGFR and the amount of proteinuria at biopsy. Model B: multiple linear regression model for eGFR decline over one year adjusted by the factors, including age, baseline eGFR, the amount of proteinuria at biopsy, and immunosuppressive therapy after biopsy. Model C: multiple linear regression model for eGFR decline over one year adjusted by the factors, including age, baseline eGFR, the amount of proteinuria at biopsy, and ARB treatment at baseline.

## Discussion

This study demonstrated the following results in patients with IgA nephropathy: (1) correlation between glomerular DNA DSBs and DNA methylation, (2) association of podocyte DNA DSBs with podocytopathic features and (3) association of glomerular γH2AX or 5mC levels with the slope of eGFR decline over one year.

We previously reported that altered DNA methylation in podocytes is involved in the pathogenesis of chronic kidney disease^21,22^, and recently suggested that impaired DNA DSB repair is associated with altered DNA methylation in podocytes^9^. Emerging evidence indicates that epigenetic mechanisms may contribute to kidney disease progression, and recent studies have successfully indicated the importance of kidney DNA methylation on renal function in humans^23,24^, although the association of DNA DSBs with DNA methylation and renal function in human kidneys has not been investigated. The present study demonstrated that glomerular DNA DSBs were positively correlated with glomerular DNA methylation in each glomerulus, which is in agreement with our previous results in murine models. A recent *in vitro* study suggested that the repair of DSBs by nonhomologous end joining (NHEJ), which is the dominant pathway of DSB repair in eukaryotes, facilitated modification of the methylation landscape in repaired genes^8^. The authors indicated that methylation patterns differed between post-repair populations and cells with uncut DNA, and that NHEJ repair could cause either hypomethylation or hypermethylation depending on the damaged region. These results suggest that the repair process itself, even if completed, alters DNA methylation. Further basic investigations of the precise mechanism of altered DNA methylation associated with DSBs and their repair may be necessary to explain the significance of altered glomerular DNA methylation.

As shown in Table 2, every 10% increase in total glomerular area according to γH2AX-positive staining or 5mC-positive staining contributed to a decrease in eGFR over one year of approximately 30 ml/min/1.73 m^2^ or 40 ml/min/1.73 m^2^, respectively. These effects were observed independently of known factors related to renal outcome, including baseline eGFR, proteinuria, and treatment with immunosuppressive agents or ARBs. Therefore, these DNA markers in glomerular cells may be good predictors of renal outcome and become novel therapeutic targets with pathways different from those of existing immunosuppressive therapies and ARB treatment.

Recent reports have shown that podocyte hypertrophy or sclerosis at the tubular pole, which are typical features associated with podocytopathies, are correlated with more proteinuria at presentation and a more rapid decline in renal function^14,25^. Therefore, we investigated podocyte DSBs using double immunostaining with WT1 and γH2AX antibody and quantitative long-distance PCR method^17^. Podocyte DNA DSBs were evaluated using long-distance PCR method in our previous study in mice^9^, and we applied this method to human samples in the present study. Podocyte DSBs evaluated by both methods were significantly correlated with podocytopathic features. Notably, podocyte DSBs were associated with the mean glomerular γH2AX levels, which suggests that the increased amount DNA DSBs in podocytes contributed to the increased amount of glomerular DNA DSBs at least in part. Further study for evaluation of DNA DSBs in other types of glomerular cells, such as endothelial cells and mesangial cells, may be necessary. The involvement of the factor mesangial hypercellularity (Oxford M0 and M1) or endothelial hypercellularity (Oxford E0 and E1) in multiple regression analysis may be useful for evaluating the contribution of DNA DSBs in proliferated mesangial cells or endothelial cells, but the M1 and E1 populations were very small in this study.

Segmental sclerosis in IgA nephropathy may result from segmental necrotizing or endocapillary inflammatory lesions, or a response to podocyte injury analogous to primary focal segmental glomerulosclerosis (FSGS)^13^. Therefore, segmental sclerosis in advanced IgA nephropathy may reflect adaptive FSGS lesions in part, which may be related to the accumulation of DNA damage, represented by increased γH2AX levels. However, Oxford classification S1 was not significantly associated with the mean glomerular γH2AX level or eGFR decline after adjustment for age, baseline eGFR and proteinuria, likely because Oxford classification S1 indicates the presence of segmental sclerosis or adhesion without quantitative evaluation of the lesions, and it does not reflect the amount of DNA DSBs in the total glomeruli.

The present study has some limitations. First, this study did not have data from long periods of time after kidney biopsy. Second, the number of subjects was small for the assessments of the involvement of clinical parameters, such as the therapy type and responsiveness to therapies, and the association of podocyte DSBs with renal outcomes. Further studies are necessary to determine the association of glomerular and podocyte DNA DSBs with renal outcomes in larger populations of patients with IgA nephropathy for longer observational periods. Third, which types of cells contributed to the increased amount of DNA DSBs in glomeruli was not clarified, but increased amounts of podocyte DSBs was associated with increased amounts of glomerular DNA DSBs, at least in part. Finally, this study focused on IgA nephropathy. However, it is necessary to investigate the importance of DNA DSBs and the association of DNA DSBs with DNA methylation and renal outcomes in other types of nephropathy.

To our knowledge, this study is the first study to demonstrate the possible association of glomerular DNA DSBs with a decline in kidney functions and glomerular DNA methylation in IgA nephropathy patients, despite these limitations. Focusing on the relationship between DNA DSBs and DNA methylation may lead us to novel approaches to understand the pathogenesis of and predict renal outcomes in chronic kidney disease patients.

## Methods

### Study population

A total of 55 individuals were diagnosed with IgA nephropathy based on kidney biopsies performed from January 1, 2015, to December 31, 2017, in Keio University Hospital. IgA nephropathy was defined immunohistologically using dominant or codominant glomerular deposits of IgA^26^. Five patients changed hospitals after biopsy or were lost to follow-up. We excluded individuals without informed consent, individuals without essential data and individuals with less than two glomeruli without global sclerosis in the biopsy samples. Patients who had IgA vasculitis nephritis or secondary IgA nephropathy were also excluded. Twenty-nine IgA nephropathy patients were ultimately included in this study. None of the patients was receiving immunosuppressive therapies at the time of biopsy. Four control samples with normal glomerular findings were obtained during the study period, from January 1, 2015, to December 31, 2017 from the patients with asymptomatic microscopic hematuria or with normal urinalysis when kidney biopsy was performed.

### Clinical evaluation and laboratory measurements

Blood pressure was measured on the right upper arm after subjects had rested at least 5 min in a sitting position in the hospital using an automatic BP-900 device with a combination of the Korotkoff sounds method and the oscillometric technique (TANITA Co. Tokyo, Japan). Blood samples were collected and immediately analyzed using an automated clinical chemical analyzer. Urinary protein excretion at biopsy was measured using 24-hour urine collection one day prior to kidney biopsy during the hospital stay. Urinary protein excretion 1 year after biopsy was calculated from the urinary protein concentration/urinary creatinine concentration at the time of the outpatient visit.

### Definitions

eGFR was calculated using the following equation: eGFR (ml/min/1.73 m^2^) = 194 × serum creatinine (−1.094) × age (−0.287) × 0.739 (if female)^27^. The slope of eGFR decline over one year was calculated by standard linear models using the data of eGFR at biopsy, 6 months and one year after biopsy as reported previously^28^. Hypertension was defined as systolic BP ≥140 mmHg and/or diastolic BP ≥90 mmHg or if the patient indicated the use of antihypertensive drugs. Diabetes at biopsy was defined in accordance with the guidelines of the American Diabetes Association as a fasting glucose concentration ≥126 mg/dl, HbA1c level ≥6.5%^29^ or the use of antihyperglycemic drugs. Two expert pathologists performed pathological diagnoses and scoring according to the Oxford Classification.

### Immunohistochemistry

The kidney samples were fixed in 10% formalin and embedded in paraffin blocks. Five-micron-thick serial sections were used for immunohistochemistry and laser microdissection. The paraffin sections were stained with periodic acid-Schiff (PAS) or γH2AX (NB100-384, Novus Biologicals, Colorado, USA) and 5-methyl cytosine (sc-56615, Santa Cruz Biotechnology, Texas, USA) antibodies followed by incubation with a peroxidase-conjugated secondary antibody and 3,3-diaminobenzidine tetrahydrochloride (DAB) staining^9^. Hematoxylin was used as a nuclear counterstain. Immunofluorescent staining was performed as previously described^21,30^ using γH2AX, WT1 (sc-7385, Santa Cruz Biotechnology, Texas, USA) and phosphorylated Ataxia Telangiectasia Mutated (pATM) (200-301-400, Rockland, PA, USA) antibodies. Images of all glomeruli without global sclerosis in the biopsy sample were acquired via microscopic examination (400x magnification), and the glomerular area was quantified using color channel analysis and pixel counting using Photoshop CC 2018 software (Adobe) and was divided by the area of the glomerular tuft, as previously described^21^.

### Laser microdissection and long distance-PCR method

Laser microdissection was performed using paraffin sections by a PALM MicroBeam IP 230 V Z microscope for laser oressure catapulting (P.A.L.M. Microlaser Technologies, Bernried, Germany) as described previously^31,32^. DNA was extracted from laser-microdissected glomerular samples using NucleoSpin Tissue (Takara Bio, Shiga, Japan). The quantitative long-distance PCR method for detecting DNA damage is based on the assumption that DNA with fewer DSB lesions will amplify to a greater extent than more damaged DNA if equal amounts of DNA from different samples are amplified under identical conditions, as described previously in mice^17,33^. We created primers to amplify an approximately 10000-bp section of the nephrin promoter region (Forward 5′-CTGCCATCAGCAACTCTCCA, Reverse 5′-CTCTGCCTCTGTTGTGCTGA, product length of 10303 bp), and we calculated the relative amount of the PCR products divided by the total glomerular area.

### Statistics

Pearson’s test was used for univariable analyses for continues variables, which are expressed as the means ± standard deviations (SD). Mann-Whitney test was used for the analysis of patients with IgA nephropathy compared to the controls. Multiple linear regression analysis was performed to evaluate the association with eGFR decline over one year, adjusted for age, eGFR and urinary protein excretion at biopsy. We confirmed each variance inflation factor (VIF) was less than 10. The presence of immunosuppressive therapy, ARB treatment, sex or presence of hypertension was also included as an adjusted factor. The significance level for all tests in this study was two-sided 5%. All statistical analyses were performed using JMP version 13 (SAS Institute Inc., Cary, NC, USA).

### Study approval

The Ethics Committee of Keio University School of Medicine approved the present study (approval number: 20180159). Written informed consent was received from participants prior to inclusion in the study. Participants were identified using a number, not their name. It was conducted in adherence with the Declaration of Helsinki. All methods were carried out in accordance with the institutional guidelines of the ethics committee at Keio University (Tokyo, Japan).

## References

[1] Lieber MR (2010). The mechanism of double-strand DNA break repair by the nonhomologous DNA end-joining pathway. Annu. Rev. Biochem..

[2] Berkovich E, Monnat RJ, Kastan MB (2007). Roles of ATM and NBS1 in chromatin structure modulation and DNA double-strand break repair. Nat. Cell Biol..

[3] Shimizu I, Yoshida Y, Suda M, Minamino T (2014). DNA damage response and metabolic disease. Cell Metab..

[4] Schupp N (2010). Aldosterone causes DNA strand breaks and chromosomal damage in renal cells, which are prevented by mineralocorticoid receptor antagonists. Horm. Metab. Res..

[5] Schmid U, Stopper H, Schweda F, Queisser N, Schupp N (2008). Angiotensin II induces DNA damage in the kidney. Cancer Res..

[6] Cuozzo C (2007). DNA damage, homology-directed repair, and DNA methylation. PLoS Genet..

[7] O’Hagan HM, Mohammad HP, Baylin SB (2008). Double strand breaks can initiate gene silencing and SIRT1-dependent onset of DNA methylation in an exogenous promoter CpG island. PLoS Genet..

[8] Allen B, Pezone A, Porcellini A, Muller MT, Masternak MM (2017). Non-homologous end joining induced alterations in DNA methylation: A source of permanent epigenetic change. Oncotarget.

[9] Hishikawa A (2019). Decreased KAT5 expression impairs DNA repair and induces altered DNA methylation in kidney podocytes. Cell Rep..

[10] Koyama A, Igarashi M, Kobayashi M (1997). Natural history and risk factors for immunoglobulin A nephropathy in Japan. Research Group on Progressive Renal Diseases. Am. J. Kidney Dis..

[11] Magistroni R, D’Agati VD, Appel GB, Kiryluk K (2015). New developments in the genetics, pathogenesis, and therapy of IgA nephropathy. Kidney Int..

[12] Working Group of the International Ig, A.N.N (2009). The Oxford classification of IgA nephropathy: rationale, clinicopathological correlations, and classification. Kidney Int..

[13] Trimarchi H (2017). Oxford Classification of IgA nephropathy 2016: an update from the IgA Nephropathy Classification Working Group. Kidney Int..

[14] Trimarchi, H. & Coppo, R. Podocytopathy in the mesangial proliferative immunoglobulin A nephropathy: new insights into the mechanisms of damage and progression. *Nephrol Dial Transplant* (2019). 10.1093/ndt/gfy413 30698804

[15] Yamada K (2015). Expression of age-related factors during the development of renal damage in patients with IgA nephropathy. Clin. Exp. Nephrol..

[16] Kinner A, Wu W, Staudt C, Iliakis G (2008). Gamma-H2AX in recognition and signaling of DNA double-strand breaks in the context of chromatin. Nucleic Acids Res..

[17] Maslov AY (2013). DNA damage in normally and prematurely aged mice. Aging Cell.

[18] Kim JA, Kruhlak M, Dotiwala F, Nussenzweig A, Haber JE (2007). Heterochromatin is refractory to gamma-H2AX modification in yeast and mammals. J. Cell Biol..

[19] Takata H (2013). Chromatin compaction protects genomic DNA from radiation damage. PLoS One.

[20] Cowell IG (2007). gammaH2AX foci form preferentially in euchromatin after ionising-radiation. PLoS One.

[21] Hayashi K (2014). KLF4-dependent epigenetic remodeling modulates podocyte phenotypes and attenuates proteinuria. J. Clin. Invest..

[22] Hayashi K (2015). Renin-angiotensin blockade resets podocyte epigenome through Kruppel-like Factor 4 and attenuates proteinuria. Kidney Int..

[23] Gluck C (2019). Kidney cytosine methylation changes improve renal function decline estimation in patients with diabetic kidney disease. Nat. Commun..

[24] Park, J. *et al*. Functional methylome analysis of human diabetic kidney disease. *JCI Insight* **4** (2019). 10.1172/jci.insight.128886 PMC6629092 31167971

[25] Bellur SS (2017). Evidence from the Oxford Classification cohort supports the clinical value of subclassification of focal segmental glomerulosclerosis in IgA nephropathy. Kidney Int..

[26] Roberts IS (2014). Pathology of IgA nephropathy. Nat. Rev. Nephrol..

[27] Matsuo S (2009). Revised equations for estimated GFR from serum creatinine in Japan. Am. J. Kidney Dis..

[28] Boucquemont J, Heinze G, Jager KJ, Oberbauer R, Leffondre K (2014). Regression methods for investigating risk factors of chronic kidney disease outcomes: the state of the art. BMC Nephrol..

[29] Diagnosis and classification of diabetes mellitus. *Diabetes Care* **33****Suppl 1**, S62–69 (2010). 10.2337/dc10-S062 PMC2797383 20042775

[30] Hayashi K (2010). Regression of glomerulosclerosis in response to transient treatment with angiotensin II blockers is attenuated by blockade of matrix metalloproteinase-2. Kidney Int..

[31] Cohen CD (2002). Laser microdissection and gene expression analysis on formaldehyde-fixed archival tissue. Kidney Int..

[32] Kohda Y, Murakami H, Moe OW, Star RA (2000). Analysis of segmental renal gene expression by laser capture microdissection. Kidney Int..

[33] Furda AM, Bess AS, Meyer JN, Van Houten B (2012). Analysis of DNA damage and repair in nuclear and mitochondrial DNA of animal cells using quantitative PCR. Methods Mol. Biol..

